# Complex of EGCG with Cu(II) Suppresses Amyloid Aggregation and Cu(II)-Induced Cytotoxicity of α-Synuclein

**DOI:** 10.3390/molecules24162940

**Published:** 2019-08-14

**Authors:** Yilong Teng, Juan Zhao, Lulu Ding, Yu Ding, Ping Zhou

**Affiliations:** 1 State Key Laboratory of Molecular Engineering of Polymers, Department of Macromolecular Science, Fudan University, Shanghai 200433, China; 2 Department of Physiology and Biophysics, School of Life Sciences, Fudan University, Shanghai 200438, China

**Keywords:** Parkinson’s disease, α-synuclein, copper, EGCG, complex

## Abstract

Accumulation of α-synuclein (α-Syn) is a remarkable pathology for Parkinson’s disease (PD), therefore clearing it is possibly a promising strategy for treating PD. Aberrant copper (Cu(II)) homeostasis and oxidative stress play critical roles in the abnormal aggregation of α-Syn in the progress of PD. It is reported that the polyphenol (−)-epi-gallocatechin gallate (EGCG) can inhibit α-Syn fibrillation and aggregation, disaggregate α-Syn mature fibrils, as well as protect α-Syn overexpressed-PC12 cells against damage. Also, previous studies have reported that EGCG can chelate many divalent metal ions. What we investigate here is whether EGCG can interfere with the Cu(II) induced fibrillation of α-Syn and protect the cell viability. In this work, on a molecular and cellulaire basis, we demonstrated that EGCG can form a Cu(II)/EGCG complex, leading to the inhibition of Cu(II)-induced conformation transition of α-Syn from random coil to β-sheet, which is a dominant structure in α-Syn fibrils and aggregates. Moreover, we found that the mixture of Cu(II) and EGCG in a molar ratio from 0.5 to 2 can efficiently inhibit this process. Furthermore, we demonstrated that in the α-Syn transduced-PC12 cells, EGCG can inhibit the overexpression and fibrillation of α-Syn in the cells, and reduce Cu(II)-induced reactive oxygen species (ROS), protecting the cells against Cu(II)-mediated toxicity.

## 1. Introduction

**Figure 1:**
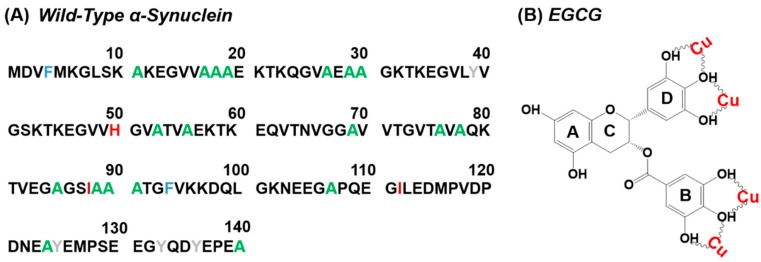
(**A**) Sequences of wild-type human α-Syn. (**B**) Structure of EGCG, and the potential Cu(II) binding sites in EGCG [19].

Misfolding of protein is involved in several serious human diseases [1,2,3,4,5]. These protein misfolding-involved diseases are characterized by the fibril aggregates or amyloid plaques present in organs, which leads to cell death [3]. For example, the fibril aggregates of α-synuclein (α-Syn), islet amyloid polypeptide (IAPP), and amyloid beta (Aβ) peptide are involved in Parkinson’s disease (PD) [6], type II diabetes (T2D) [7], and Alzheimer’s disease (AD) [8], respectively. PD is one of the neurodegenerative movement disorders characterized by impairment of movement function [9]. The pathophysiological characteristics of PD include degeneration and death of dopaminergic neurons and the accumulation of intracellular inclusions designated as Lewy body [10]. Amyloid-like α-Syn fibrils are the primary structural component of Lewy body [11]. The aggregation of α-Syn is thought to play an important role on PD [12]. Under normal physiological status, α-Syn is an intrinsically unfolded protein (Figure 1A), which comprises of 140 amino acids distributed in three regions: (i) the amphipathic N-terminus; (ii) the highly hydrophobic self-aggregating sequence known as NAC (non-Aβ component); and (iii) the acidic C-terminal region [13]. This natively disordered protein can convert to mature fibrils with β-sheet conformation through an aggregation pathway relevant to the oligomer species, as the intermediates [14]. 

Epidemiological evidence indicated the risk of PD upon the occupational exposure to heavy metals [15]. In brains of PD patients, copper, iron, zinc, and aluminum in high concentrations were detected in the cerebrospinal fluid (CSF) or the substantia nigra [16,17]. The oxidative stress is considered to be a pathogenic factor for PD [18]. The redox-active metal ions concentrated in substantia nigra could result in oxidative conditions more favorable for radical species production [19]. It was also proposed that metal ions may promote the fibrillation and aggregation of α-Syn, possibly through the secondary structural changes of α-Syn caused by direct interactions between protein and metal ions [20]. Here, it is worth noting that copper (Cu(II)) is a particular agent, which, in addition to accelerating fibrillation and aggregation of protein, is also an oxidant. 

Cu(II) is an essential metal with an average concentration of 1.4~2.1 mg/kg in healthy individuals, and it is readily absorbed from the diet through the small intestine (~2 mg/day) [21]. However, accumulation of Cu(II) is observed in brain areas linked to both PD [22] and AD [23]. High concentration of Cu(II) is present in the CSF of PD patients. In particular, Cu concentration is almost two or three folds higher than control levels (10.2 ng/g) [24]. Therefore, it is important to develop an appropriate Cu(II) ion chelator for PD therapy.

Many studies suggest that catechins are potential drugs for the neuroprotection [25]. The primary flavonoid phytochemical compounds present in green tea are catechins, and epigallocatechin gallate (EGCG) is a dominant component [26]. In hippocampal neurons, tea polyphenols show a protective effect against ischemic insult, and EGCG can attenuate neurotoxicity induced by β-amyloid [27]. It has been reported that EGCG (Figure 1B) possess the abilities of chelation of divalent metal ions, antioxidantion, and anti-inflammation, to protect neurons from death in a wide array of cellular and animal models of neurological disorders [28]. EGCG can directly scavenge reactive oxygen species (ROS) to exert remarkably antioxidant effects [19]. In addition, EGCG can inhibit α-Syn conformation transition from random coil to β-sheet, and convert large, mature α-Syn fibrils into smaller, amorphous protein aggregates that are nontoxic to cells [29]. These findings suggest that EGCG is a potential agent for PD and AD treatment.

Cu(II) and EGCG were both known to bind α-Syn and modulate its fibrillation [20,29], but the effect of EGCG on Cu(II)-induced α-Syn aggregation, as well as their influences on α-Syn expression in cells, were not well studied. Here, we investigated the effects of Cu(II), EGCG, and their mixture on α-Syn aggregation on molecular and cellular levels by spectroscopic, microscopic, and molecular biologic methods.

## 2. Results

### 2.1. Prevention of Cu(II)-Induced α-Syn Amyloid Fibrillation by EGCG

**Figure 2:**
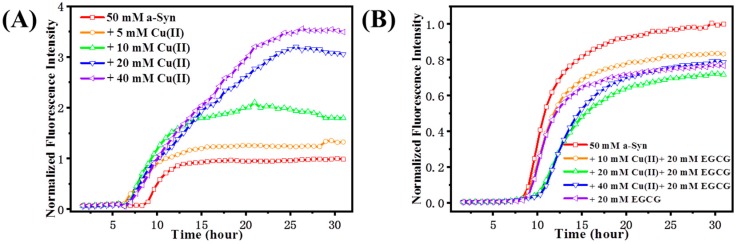
ThT fluorescence of α-Syn with Cu(II) and EGCG presence. (**A**) The fibrillation kinetics of 50 μM α-Syn in the absence or presence of 10, 20, and 40 μM Cu(II), respectively. (**B**) The fibrillation kinetics of 50 μM α-Syn incubated with Cu(II) concentration of 0, 10, 20, and 40 μM and EGCG constant concentration of 20 μM. The maximum fluorescence intensity of control group (50 μM α-Syn) was normalized as 1.0, the intensities for other groups were referred to it.

ThT is a commonly used assay for the β-sheet amyloid fibrillation induced by protein misfolding. Specifically, binding of ThT dye to cross-beta architecture induces an enhanced fluorescence intensity, which sometimes accompanies a redshift in the fluorescence wavelength. Changes in the ThT fluorescence intensities are used to indicate an extent of amyloid fibril aggregates [30]. The kinetics of fibrillation of α-Syn from helix/random coil to β-sheet conformational transition was monitored by ThT fluorescence assay (Figure 2). Figure 2A shows the time-course profile of Cu(II)-induced α-Syn conformational transition. Upon excitation of the ThT-stained samples at 450 nm, the control sample of α-Syn displayed the lowest intensity in fluorescence, while the addition of Cu(II) resulted in a significant increase in the fluorescence intensity, which promoted the amyloid aggregation as the increase of molar ratio of Cu(II) to α-Syn. Furthermore, we investigated the effect of EGGC on the Cu(II) induced α-Syn aggregation (Figure 2B). α-Syn incubated with 20 μM EGCG led to a decrease of β-sheet content, as demonstrated by ThT fluorescence intensity declining dramatically, and the lag phase was elongated with the addition of the mixture of EGCG and Cu(II), compared with that merely treated with Cu(II), which indicates that EGCG prevented Cu(II)-induced amyloid fibrillation of α-Syn. As the ratio of Cu(II) to EGCG was increased from 0.5 to 2, the lag time was delayed, and β-sheet formation was inhibited. When the mixtures of Cu(II) and EGCG in molar ratios of 1:1 and 2:1 were added to the sample, the ThT fluorescence intensity even dropped significantly more than that of group in the presence of 20 μM EGCG. These results suggest that EGCG interacts with Cu(II), forming a complex which played a vital role in inhibiting the aggregation of α-Syn. 

**Figure 3:**
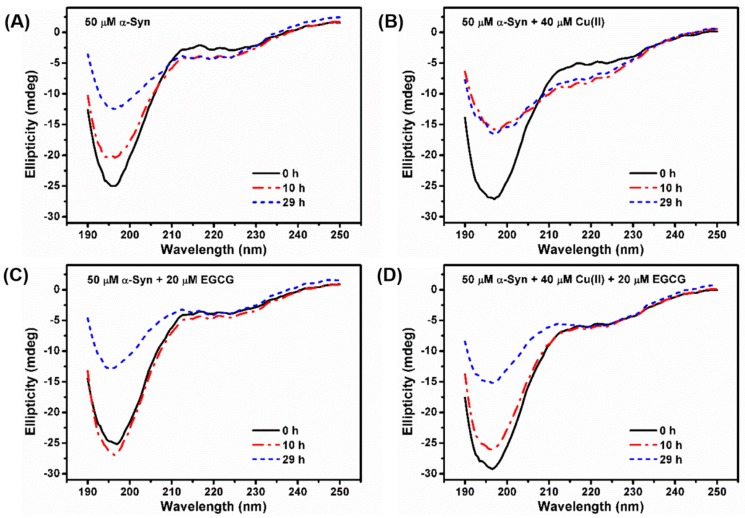
CD spectra of 50 μM α-Syn incubated in the absence (**A**) or presence of (**B**) 40 μM Cu(II), (**C**) 20 μM EGCG, (**D**) the mixture of 20 μM EGCG and 40 μM Cu(II) after incubation for 0 h (line in black), 10 h (dash in red), and 29 h (dot in blue).

Protein secondary structure can also be monitored by far-UV CD. At the initial state, α-Syn shows a typical spectrum of an essentially unfolded protein with characteristic negative bands at 198 nm and 222 nm (Figure 3A). When α-Syn was incubated for a while, changes in the shape of its spectrum were observed. A decrease at 198 nm was accompanied by an increase in negative intensity around 222 nm, reflecting the formation of the ordered β-sheet secondary structure. Larger changes in the secondary structure were observed when α-Syn was incubated with 40 μM Cu(II) for up to 10 h (Figure 3B), with signal intensity of the negative peak around 222 nm increased, suggesting that Cu(II) promoted the conformation transition of α-Syn from random coil to β-sheet structure. However, with the addition of 20 μM EGCG (Figure 3C), the negative ellipticity at 222 nm was changed slowly, compared to that of α-Syn alone, indicating that the EGCG inhibited the β-sheet conformation. Moreover, the CD spectrum of α-Syn treated with a mixture of 40 μM Cu(II) and 20 μM EGCG was similar to that of the sample in the presence of 20 μM EGCG (Figure 3D). These findings indicated that EGCG hindered Cu(II)-induced conformation transition of α-Syn from random coil to β-sheet state, consistent with the ThT fluorescence results.

**Figure 4:**
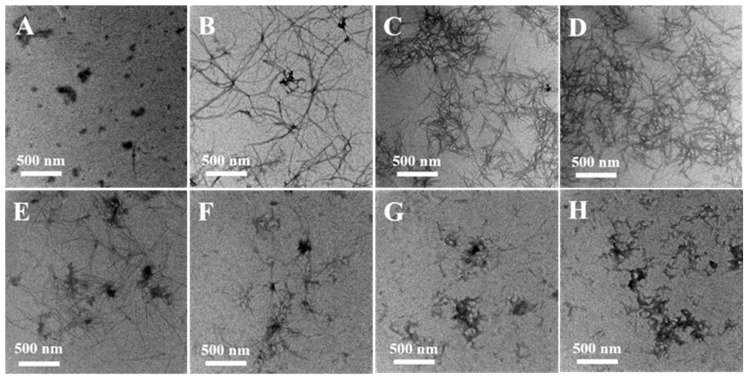
TEM analysis of morphologies of α-Syn aggregates. 50 μM α-Syn incubated alone for 0 h (**A**), 29 h (**B**). 50 μM α-Syn incubated in the presence of (**C**) 20 μM Cu(II), (**D**) 40 μM Cu(II), (**E**) 20 μM EGCG, (**F**) the mixture of 10 μM Cu(II) and 20 μM EGCG, (**G**) the mixture of 20 μM Cu(II) and 20 μM EGCG, and (**H**) a mixture of 40 μM Cu(II) and 20 μM EGCG, respectively.

The effects of EGCG on Cu(II)-induced fibril formation and morphology were studied further by TEM (Figure 4). After being incubated for 28 h, a negatively stained sample of α-Syn contained the elongated and interlaced fibers (Figure 4B), instead, a few of the compact small aggregates were observed in the sample at the beginning of incubation (Figure 4A). When α-Syn was incubated with 20 μM (Figure 4C) and 40 μM (Figure 4D) Cu(II), lots of fibrils were formed. Incubation of α-Syn with EGCG did not result in further fibril formation detected by TEM (Figure 4E), which indicated that EGCG controlled the fibrillation process of α-Syn. Furthermore, the mixture of various ratios of Cu(II) and EGCG incubated with α-Syn was also observed by TEM, and numerous large amorphous aggregates were found (Figure 4F–H), instead of fibrils formed in the sample only treated with Cu(II). These observations are consistent with the results of the ThT fluorescence and CD analysis, which suggest that EGCG may interact with Cu(II), reducing the Cu(II)-induced fibrillation of α-Syn. The ordered β-sheet fibrils of α-Syn result in the high ThT fluorescence, but the amorphous aggregates of α-Syn do not.

### 2.2. Interaction of Cu(II), EGCG, and Cu(II)/EGCG with α-Syn Demonstrated by ^1^H-NMR

**Figure 5:**
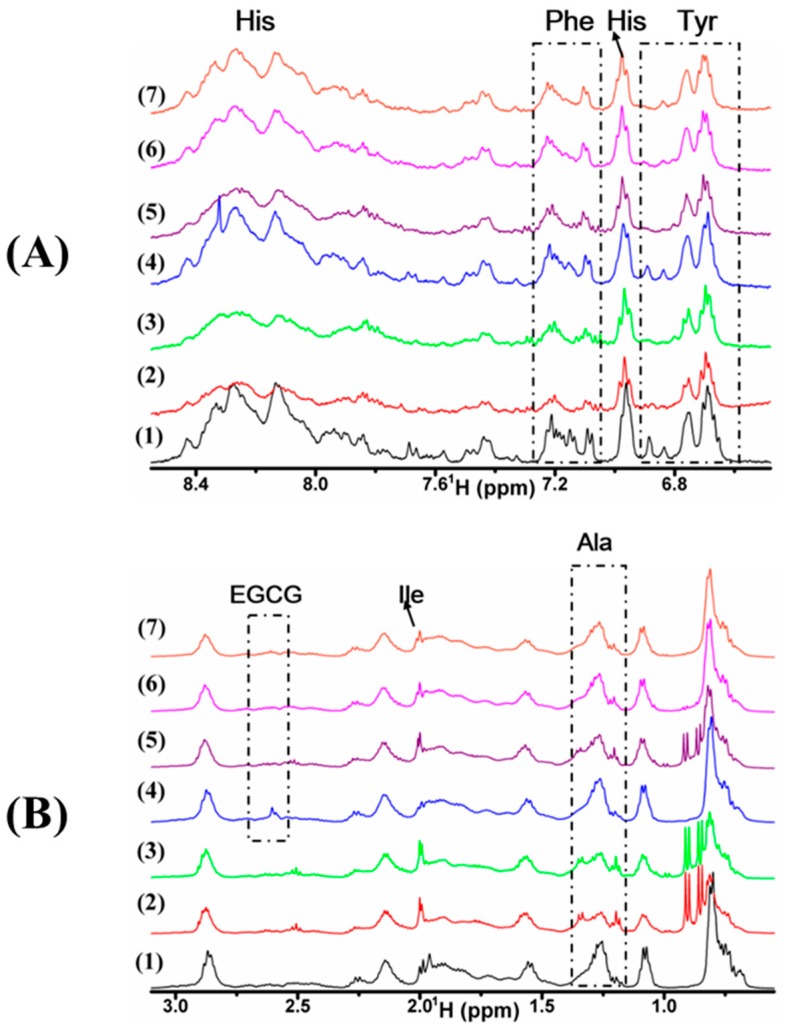
^1^H-NMR of α-Syn treated with Cu(II), EGCG, or their mixture. (**A**) The aromatic region of α-Syn within 6.55 and 8.65 ppm; (**B**) high field region of α-Syn and EGCG within 0.55 and 3.15 ppm. Samples are 50 μM α-Syn incubated for 0 h (1) and 29 h (2) alone, in the presence of 40 μM Cu(II) (3), 20 μM EGCG (4), the mixture of 10 (5), 20 (6), 40 (7) μM Cu(II) with 20 μM EGCG, respectively, for 58 h in PBS buffer (20 mM, pH 7.4).

For each sample of α-Syn, ^1^H-NMR spectrum was recorded using a water suppression pulse, and selectively analyzed for the aromatic region from 6.55 to 8.65 ppm (Figure 5A), and the high field region from 0.55 to 3.15 ppm (Figure 5B). The spectral linewidth of α-Syn was incubated solely for 24 h (Figure 5A(2)) was broadened apparently, compared with that without incubation (Figure 5A(1)), which suggests that the rigid aggregates of α-Syn were formed. The spectrum of α-Syn incubated with Cu(II) (Figure 5A(3)) is similar to that of α-Syn incubated solely. By contrast, when EGCG was added to α-Syn, the linewidths (Figure 5A(4)) were similar to that of α-Syn without incubation (Figure 5A(1)), which indicates that EGCG restrained the aggregation. Furthermore, to explore the effect of Cu(II)/EGCG mixture on α-Syn fibrillation, we recorded ^1^H-NMR spectrum of 50 µM α-Syn in the presence of the mixture of Cu(II) and EGCG in molar ratio at 0.5 (Figure 5A(5)), 1.0 (Figure 5A(6)), and 2.0 (Figure 5A(7)). The line broadening of α-Syn incubated with Cu(II)/EGCG mixture was smaller (the peak of Ala at 1.25 ppm in Figure 5B(5–7)) than that incubated with Cu(II) (Figure 5A(3)), indicating that EGCG blocked Cu(II) binding to α-Syn. Additionally, when EGCG coexisted with Cu(II), the peak of EGCG at 2.60 ppm notably disappeared, which suggests that EGCG interacted with Cu(II). These results indicate that the complex of Cu(II)/EGCG inhibits the aggregation of α-Syn. From Figure 5A(3), the double peak of Tyr at 6.85 ppm remarkably disappeared, which suggests that Tyr might be involved in the interaction with Cu(II). Nevertheless, Figure 5B(4) shows that the peak of Ile (δ 1.95 ppm) was broadened, which suggests that Ile might be involved in the interaction with EGCG. We supposed that the Cu(II)/EGCG complex might bind to Tyr to prevent the fibrillation rather than to Ile, which is different from EGCG. Comparing the spectra with the different ratio of Cu(II) to EGCG, we found that the complex of Cu(II)/EGCG complex in molar ratio at 1.0 and 2.0 effectively inhibited the process of aggregation. 

### 2.3. Suppression of the Cu(II)-Induced Overexpression and Aggregation of α-Syn by EGCG in the Transduced PC12 Cells

**Figure 6:**
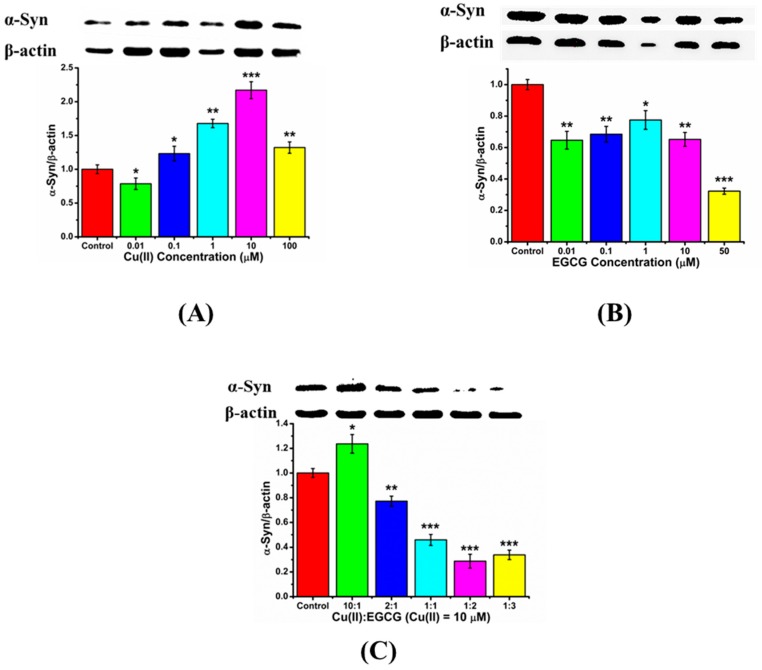
Western blot analyses for the α-Syn expression in the transduced PC12cells. The β-actin was used as an endogenous quantitative reference. Cells were treated with (**A**) Cu(II) (0, 0.01, 0.1, 1, 10, and 100 μM), (**B**) EGCG (0, 0.01, 0.1, 1, 10, and 50 μM), and (**C**) the mixture of 10 μM Cu(II) with EGCG in ratio of Cu(II)/EGCG as 10:1, 2:1, 1:1, 1:2, and 1:3 for 48 h, respectively. Error bars = SD, *n* = 3; *: *p* < 0.05, **: *p* < 0.01, ***: *p* < 0.001 compared with the control group (protein only).

Here, Western blot was used to quantitatively analyze the protein expression. We investigated the endogenous α-Syn expression in the transduced PC12 cells influenced by Cu(II), EGCG, and their mixture. The expression level of α-Syn was increased with Cu(II) concentration from 0.01~10 μM, however, it decreased when Cu(II) concentration was 100 μM (Figure 6A). The positive charged Cu(II) ion as a bridge might link the electron-enriched amino acids, such as His and Tyr, between α-Syn chains, leading to the α-Syn gathering and aggregation. The α-Syn aggregation induced by Cu(II) would further intrigue its overexpression in cells [31]. However, much high concentration of Cu(II), such as 100 μM, may surround the proteins with many positive charges, and form the electrostatic repulsion between protein chains, leading to the inhibition of the β-sheet aggregation [32], therefore resulting in the less expression of α-Syn. From Figure 6B, EGCG reduced the expression of α-Syn in the cells. Further, Figure 6C shows that the mixtures of Cu(II) and EGCG resulted in a remarkable down-regulation of α-Syn expression. We found that the mixture of Cu(II) and EGCG in a ratio from 0.5 to 2 can down-regulate the α-Syn expression. These results indicate that EGCG suppressed the Cu(II)-induced α-Syn overexpression in the transduced PC12 cells.

**Figure 7:**
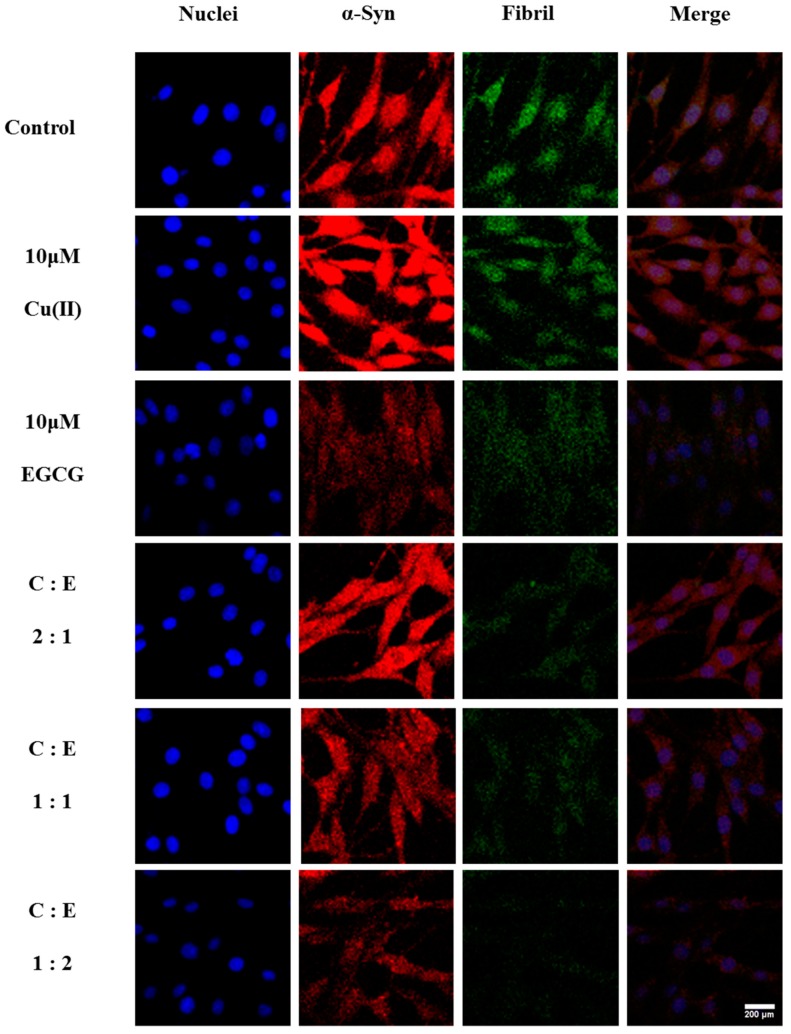
LSCM images of α-Syn aggregates in the transduced PC12 cells treated without and with 10 μM Cu(II), 10 μM EGCG, or the mixture of 10 μM Cu(II) (symbol of C) with EGCG (symbol of E) in ratio of Cu(II)/EGCG (C:E) as 2:1, 1:1, and 1:2 for 48 h, respectively. α-Syn in the red, α-Syn fibrils in the ThT green, and the cell nuclei in the DAPI blue. Scale bars = 200 μm. The pictures shown are representative of three individual experiments.

To further verify the effect of EGCG on Cu(II)-induced α-Syn overexpression in transduced PC12 cells, α-Syn expression and immunoreactivity were observed in the cells using LSCM, and ThT fluorescence assay was employed to investigate the ordered α-Syn fibrillation. 10 μM Cu(II) significantly increased the immunoreactivity and aggregation of α-Syn, compared with that of α-Syn alone (Figure 7). However, 10 μM EGCG treatment significantly reduced α-Syn expression and aggregation. Furthermore, the Cu(II)-induced overexpression and aggregation of α-Syn were inhibited by EGCG treatment. Interestingly, when molar ratio of Cu(II) to EGCG at 1:1 and 1:2, the mixture inhibits the aggregation and fibrillation of α-Syn more efficiently than EGCG alone, consistent with the α-Syn expression determined by Western blot. These observations indicate that EGCG protects the cells against Cu(II)-induced neurotoxicity through the modulation of α-Syn expression and aggregation.

### 2.4. Protection of Cell Activity against Cu(II)-Induced Toxicity in the α-Syn Transduced PC12 Cells by EGCG

**Figure 8:**
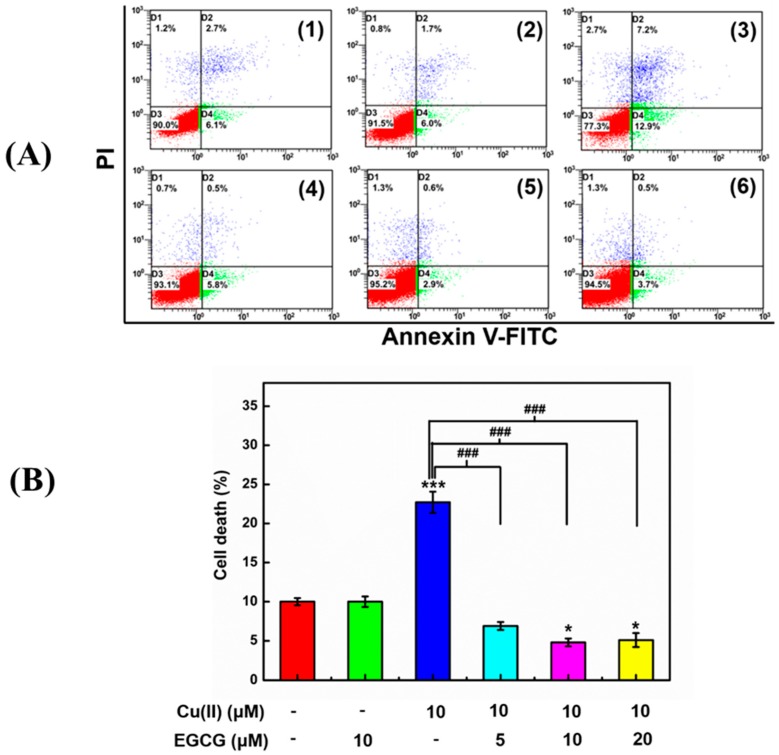
EGCG effect on the cells activity against Cu(II)-induced cytotoxicity in the transduced PC12 cells. (**A**) FACS analysis of the cells treated without (**1**) (control) and with 10 μM EGCG (**2**), 10 μM Cu(II) (**3**), the mixture of 10 μM Cu(II) with 5 (**4**), 10 (**5**), and 20 (**6**) μM EGCG for 48 h, respectively. (**B**) The cell apoptotic percentage in D2 and D4 phase together with FACS determined by PI and Annexin V-FITC fluorescence intensity upon various indicated treatments. Error bars = SD, *n* = 3; *: *p* < 0.05, ***: *p* < 0.001 vs. control group; ###: *p* < 0.001 vs. [Cu(II)] = 10 μM.

To evaluate EGCG protecting the cells activity against Cu(II)-induced apoptotic for the α-Syn transduced PC12 cells, the annexin V/PI double fluorescence staining and flow cytometry (FACS) were applied (Figure 8). The early apoptotic cells are probed by annexin V conjugated to fluorescein isothiocyanate (annexin V-FITC) which interact with the phosphatidylserine externalized in the apoptotic cells. The annexin V-FITC fluorescence was analyzed by FL1 (Ex: 488 nm, Em: 530 nm). The late-apoptotic cells are probed by PI uptake into the broken plasma membrane of apoptotic cells and interaction with DNA in cell nucleus. The complex of PI and DNA was detected using FL-3 (Ex: 488 nm; Em: 695 nm). The early apoptotic cells locate in D4 phase of flow cytometry in Figure 8(A) with both positive annexin V-FITC and negative PI fluorescence. The late-apoptotic and nonviable cells locate in D2 phase with both positive annexin V and PI fluorescence. The necrotic cells, which are induced by the improper mechanical force, locate in D1 phase with both negative annexin V-FITC and positive PI fluorescence. The viable cells locate in D3 phase with both negative annexin V-FITC and negative PI fluorescence. The apoptotic cells are represented by a percentage determined by the cells in D2 and D4 together. The α-Syn transduced PC12 cells treated with 10 μM Cu(II) showed a significantly increased apoptotic cells percentage (Figure 8B) from early apoptotic cells and late-apoptotic and nonviable cells (Figure 8A(3)), compared with the control cells (Figure 8A(1) and Figure 8B). However, 10 μM EGCG (Figure 8A(2) and Figure 8B) had no effects on the apoptotic cells percentage, which means that EGCG is non-toxic for the transduced PC12 cells. Interestingly, the mixture of 10 μM Cu(II) and 5 μM (Figure 8A(4) and Figure 6B), 10 μM (Figure 8A(5) and Figure 8B), and 20 μM (Figure 8A(6) and Figure 8B) EGCG decreased the cell’s apoptotic percentage. These results manifested that EGCG protects the transduced PC12 cells against Cu(II)-mediated toxicity. 

### 2.5. Inhibition of Cu(II)-Induced ROS by EGCG in α-Syn Transduced PC12 Cells

**Figure 9:**
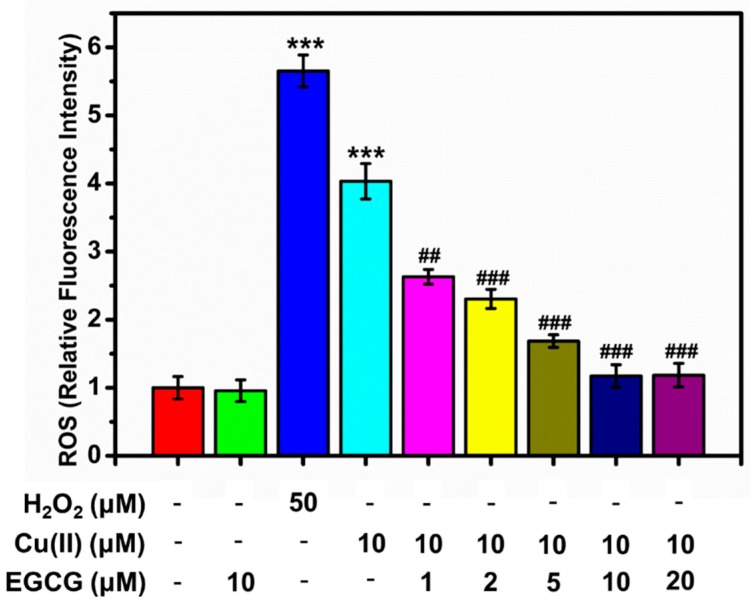
ROS in the α-Syn transduced PC12cells treated by H_2_O_2_ for 15 min, Cu(II), and EGCG for 24 h. Error bars = SD, *n* = 3; ***: *p* < 0.001 vs. control group; ##: *p* < 0.01, ###: *p* < 0.001 vs. [Cu(II)] = 10 μM.

For the analysis of intracellular ROS by FACS, the oxidation-sensitive probe of DCFH-DA was used. We studied the effect of Cu(II), EGCG, and the mixture of Cu(II) and EGCG on the intracellular ROS (Figure 9). ROS levels were studied for the series of α-Syn transduced PC12 cells samples: α-Syn transduced PC12 cells (control group), and the one treated by 50 μM H_2_O_2_ for 15 min, 10 μM Cu(II), 10 μM EGCG, and 10 μM Cu(II) mixture with 5, 10, and 20 μM EGCG for 24 h. From Figure 9, the production of ROS was considerably increased by 50 μM H_2_O_2_, as well as 10 μM Cu(II). 10 μM EGCG alone did not induce the ROS production. Cu(II) and EGCG mixture markedly reduced the ROS formation, compared with the group treated with 50 μM H_2_O_2_ as well as 10 μM Cu(II). Additionally, as molar ratio of Cu(II) to EGCG went from 5 to 0.5, the amount of ROS induced by 10 μM Cu(II) was significantly inhibited. Thus, it was concluded that EGCG inhibited the Cu(II)-induced generation of ROS in the α-Syn transduced PC12 cells.

## 3. Discussion

Metal ions play crucial roles in neural transmission, oxygen transport, and synthesis/metabolism of neurotransmitters in the brain [33,34]. On the other hand, the dysregulation of metal ions can also result in oxidative stress and disorders. For instance, Cu(II) is involved in many metal-mediated diseases [17]. Cu(II)-enhanced α-Syn amyloid formation is a direct consequence of Cu(II)-α-Syn complex formation [35]. Nevertheless, the mechanism of metal ions in promoting α-Syn neurotoxicity still remains unclear.

There is extensive epidemiological evidence for green tea health benefits. EGCG extracted from green tea can chelate metal ions to form the complex which can be used for the treatment of neurodegenerative disorders [36,37]. EGCG interacts with α-Syn competitively when Cu(II) is present, resulting in the inhibition of α-Syn fibrillation induced by Cu(II). In our study, the complex of Cu(II)/EGCG can inhibit α-Syn fibrillation, possibly because of its large stereo size and interaction with His and Tyr residue in α-Syn. In addition, EGCG acting as radical scavenger has also been attended.

### 3.1. EGCG Coordinates Cu(II) to Form a Cu(II)/EGCG Complex and Hinders α-Syn Conformation Transition

Significant efforts have been devoted to investigate the Cu(II)-binding to α-Syn. α-Syn is able to bind five or more Cu(II) with a *K*_d_ = 4~6 × 10^−5^ M [38]. Initially, the primary Cu(II)-binding site involves His50 as the anchoring residue with nitrogen/oxygen donor atoms in a square planar [39]. The acidic C-terminus of the protein coordinate the second Cu(II) equivalent with a 300-fold lower affinity [39]. Studies have demonstrated that the Cu(II) binding site with the highest affinity is located at the first few N-terminal residues of α-Syn, with a binding constant of about 100 nM [40,41,42]. The presence of Cu(II) significantly enhances the content of the β-like structure in monomeric α-Syn [43], leading to the aggregation of α-Syn upon Cu(II) binding [44].

Many agents have been used to chelate Cu(II) in blood. Ammonium tetrathiomolybdate [45] and penicillamine [46] are well-known chelators of Cu(II) for the antiangiogenic therapy, but their side effects were also severe [47]. Thus, developing safe and efficient compounds from dietary constituents to chelate Cu(II) has been a hot topic. EGCG is one of those compounds [48]. The stoichiometry of Cu(II)/EGCG complex was reported in the molar ratio of 2:1 [49], which is similar to Fe(III)/EGCG complex in previous study [50]. The chelating sites of EGCG are 3,4-dihydroxy groups in the B ring and D ring (Figure 1B) [19,51].

In our study, as shown in the NMR spectra of Figure 5B, EGCG interacted with Cu(II), and when the stoichiometry of Cu(II) to EGCG was 2:1, the β-sheet content was less than that in the presence of EGCG alone, indicating that the Cu(II)/EGCG complex is more efficacious than EGCG to inhibit the aggregates of α-Syn. The Cu(II)/EGCG complex binds to Tyr to prevent the fibrillation. Similar results were reported in our previous study for Fe(III)/EGCG complex. EGCG can attenuate the Fe(III)-induced conformational transition of alpha-synuclein [52]. Fe(III) interacts with the amino residue of Tyr and Ala of α-Syn, then accelerates the fibrillation of α-Syn. EGCG formed the stable complexes with Cu(II) by gallate moiety, which increases the steric hindrance of α-Syn interaction with itself, inhibiting the aggregation of α-Syn.

### 3.2. EGCG Inhibits the Cu(II)-Induced ROS in the Cells

The toxicity of Cu(II) is mostly associated with oxidative damage, inducing the production of ROS, which attacks DNA and biological macromolecules. Copper generates OH^∙^ radical in a Fenton-like reaction with H_2_O_2_ [53], and the OH^∙^ radical can damage DNA [54]. Previous study has demonstrated that Cu(II)/α-Syn complex is capable of redox cycling, resulting in the generation of oxygen radicals, which leads to oxidative stress and chemical modification of α-Syn [55]. It was also shown that Cu(II) binds to α-Syn with high affinity, playing a role in Cu(II) metabolism in vivo, and increasing the neurotoxicity of α-Syn oligomers [56]. Cu(II)-loaded cytotoxic oligomers of α-Syn have been isolated and show a unique stellate morphology [57]. Therefore, the agent which can remove Cu(II) from α-Syn and also savage ROS would be a potential for treatment of PD.

EGCG, a potent antioxidant, can form a complex with metal ions and thus inhibit the metal ion effects on the ROS production. The polyphenols from green tea have been proved to have the neuroprotective function in vivo through the interaction with antioxidant protective enzymes, such as superoxide dismutase and catalase, to eliminate the activity of these two oxygen radical species [57].

In this work, we also demonstrated that EGCG can protect α-Syn transduced PC12 cells against α-Syn-induced cells damage by reducing α-Syn overexpression, fibrillation, and ROS. Also, vice versa, EGCG inhibit Cu(II)-induced ROS generation, leading to the inhibition of the overexpression and fibrillation of α-Syn in the cells. Moreover, the mixture of Cu and EGCG shows a cytoprotection better than EGCG alone. The results are similar to that of the Fe(III)/EGCG complex studied in our previous work [52]. EGCG can significantly inhibit the Fe(III)-induced fibrillation of α-Syn by chelating Fe(III) and protect α-Syn-transduced PC12 cells against the toxicity induced by ROS and β-sheet-enriched α-Syn fibrils [52]. On the basis of studies for molecules and cells, we suggest that EGCG protect the α-Syn transduced PC12 cells against the Cu(II)-mediated toxicity through several ways: (i) coordinate with Cu(II), (ii) eliminate Cu(II)-induced ROS, and (iii) form a Cu(II)/EGCG complex, which effectively decreases the overexpression and fibrillation of α-Syn.

## 4. Materials and Methods

EGCG, thioflavin T (ThT), D_2_O, 2′,7′-Dichlorofluorescin diacetate (DCFH-DA), tris-hydroxymethyl aminomethane (Tris), and phenylmethanesulfonyl fluoride (PMSF) were purchased from Sigma and Aldrich, USA. Horse serum (HS), fetal bovine serum (FBS), phosphate buffered saline (PBS), Dulbecco’s modified Eagle’s medium (DMEM), l-glutamine and penicillin/streptomycin antibiotics were purchased from Gibco, USA. Glycerol, imidazole, cupric chloride (CuCl_2_∙H_2_O), hydrochloric acid (HCl), KH_2_PO_4_, Na_2_HPO_4_·12H_2_O, KCl, and NaCl were sourced from Sinopharm, China. Annexin V-FITC (fluorescein isothiocyanate, FITC) and propidium podide (PI) were purchased from Dojindo Laboratories. RIPA buffer, 0.01% poly-l-lysine, goat serum, and 4′,6-diamidino-2-phenylindole (DAPI) were obtained from Songon Biotech, China. Antifade medium, paraformaldehyde, and Triton X-100 were purchased from Beyotime, China. The PC12 cell was obtained from Shanghai Institutes for Biological Sciences, China. 

### 4.1. α-Syn Expression and Purification

The expression and purification of α-Syn was described in our previous report [52]. Briefly, the α-Syn gene was amplified by PCR and cloned into the pT7N10C6 vector, which was reconstructed from the commercialized pET15b vector (Novagen, Darmstadt, Germany) with N-terminal 10-histidine tag and TEV protease (Tobacco Etch Virus protease) cleavage site. The expression plasmid pT7N10C6-AS was then introduced into Escherichia coli BL21 (DE3) pLsyS, within which the recombinant AS protein was expressed using the auto-induction system at an initial temperature of 37 °C. When the bacteria were cultured until the optical density (O.D.) = 0.8 (λ = 600 nm), they were incubated at 16 °C for 20 h for the transferred reaction. The bacteria were collected by centrifugation (6000× *g*, 4 °C, 15 min) and suspended in 50 mM Tris-HCl buffer, pH 8.5, with 150 mM NaCl and 5% glycerol, followed by centrifugation (20,000× *g*, 4 °C, and 60 min). The protein in supernatants were purified by HisTrap HP column (GE Healthcare, Fairfield, CT, USA) and eluted with 50 mM Tris-HCl buffer, pH 8.5, containing 150 mM NaCl, 5% glycerol and 140 mM imidazole. Then the His_10_ tag on the α-Syn was removed by incubation with His-tagged TEV protease overnight and separated on a HisTrap HP column (GE Healthcare). The α-Syn protein was further purified by Superdex 75 Increase 10/300 GL size exclusive column (GE Healthcare). Finally, the protein was exchanged into Tris-HCl buffer by ultrafiltration. 

### 4.2. ThT Fluorescence Assays 

α-Syn fibrillation was monitored by recording ThT fluorescence intensity every 10 min for 29 h at 37 °C, using a Bio Tek Synergy H1 plate reader (Bio Tek, Winooski, VT, USA), which was described in our previous report [29,52]. Briefly, the lyophilized drying α-Syn was dissolved in Tris-HCl buffer (20 mM, pH 7.4). The given concentrations of CuCl_2_ or EGCG and ThT from stock solution were added to each sample, and the final solution contained 50 µM protein, and 10 mM ThT. 100 µL of mixture solution was placed in each well for ThT fluorescence assay at the excitation wavelength of 450 nm and the emission wavelength of 485 nm. Samples were incubated at 37 °C in 96-well black plates (Corning, New York, NY, USA). Plates were continuously shaken at 731 rpm for the entire period of study at a rotation diameter of 2 mm. The profiles obtained from kinetic analysis were fitted using the Origin 7.5 software (OriginLab, Northampton, MS, USA). 

### 4.3. CD Analysis

CD (circular dichroism) spectra for samples in Tris-HCl buffer (20 mM, pH = 7.4) were recorded at 25 °C with a JASCO J-1500 circular dichroism spectrophotometer (JASCO, JAP) in a 1 mm path length quartz cuvette at the scanning range from 185 to 260 nm. To eliminate the contributions from the buffer, we subtracted the baseline (20 mM phosphate buffer alone) from the corresponding spectrum. Data were averaged for three scans at a speed of 50 nm/min, collected in 1 nm steps.

### 4.4. Transmission Electron Microscopy (TEM)

5 μL of the protein solution was collected from the solution of ThT measurements and diluted to 25 μg/mL solution with Tris buffer quickly. Then, 10 μL of the diluted sample was blotted on a Cu(II) grid (carbon-coated Formvar 300 mesh, Electron Microscopy Sciences, Hatfield, PA, USA), and stained with 2% (*w/v*) uranyl acetate for 20 min. The grids were allowed to dry thoroughly. Images were acquired using a Tecnai G^2^ 20 TWIN transmission electron microscope (FEI, Hillsboro, OR, USA) at 200 kV. 

### 4.5. ^1^H-NMR Spectroscopy

α-Syn samples were dissolved in Tris-HCl (20 mM, pH 7.4), and then mixed with CuCl_2_ or EGCG aqueous solutions to obtain the protein concentration of 50 μM. The samples were incubated shakily at 37 °C for 58 h, and then D_2_O was added in the ratio of 90% H_2_O/10% D_2_O for ^1^H-NMR measurement at 25 °C. ^1^H-NMR spectra were recorded on an AVANCE III HD 500 MHz spectrometer (Bruker, Karlsuhe, Germany). Water signal was suppressed using the WATER-GATE pulse sequence [58].

### 4.6. Cell Culture

PC12 cell is a cell line that originated from a pheochromocytoma of the rat adrenal medulla. As α-Syn are not well expressed naturally in PC12 cells [59], we transduced α-Syn gene into the cells for the study, which was described in previous work [60]. Briefly, α-Syn gene was amplified by PCR and cloned into pDrive vector (Invitrogen, Waltham, CA, USA). α-Synuclein in pDrive was subcloned in the PatI-XhoI sites of a pLenti6/V5 expression vector (Invitrogen, USA) at a downstream of a cytomegalovirus promoter. The orientation and sequence of the construct was confirmed by restriction analysis and DNA sequencing. Then the lentiviral stocks were produced by cotransfecting the optimized packaging plasmid, and pLenti expressing construct was mixed into the 293T cell line according to the manufacturer’ protocols (The ViraPowerTM Lentiviral Expression System, Invitrogen, USA), as described previously [61]. After α-synuclein in lentiviral stocks was transduced into PC12 cell line, the individual stably transduced colony was subsequently selected in the presence of blasticidin (Invitrogen, USA). Finally, the expression level of a-synuclein was assessed by Western blot. Transduced PC12 cells were grown in DMEM containing 6.7% HS, 3.3% FBS, 1% l-glutamine (3.6 mM), and 1% penicillin/streptomycin antibiotics in 5% CO_2_ at 37 °C. Cells were harvested from flasks and plated in polystyrene plates (Corning, New York, NY, USA), and incubated at 37 °C for 24 h to attach the plates, and then the medium was removed from each well. Cells were treated with Cu(II), EGCG, or their mixture, respectively, in fresh medium (DMEM containing 1.0% HS, 0.5% FBS, 1% l-glutamine (3.6 mM)), and 1% penicillin/streptomycin antibiotics for efficiently up-taking EGCG and Cu(II) in the medium. The cells were then incubated for an additional 48 h at 37 °C.

### 4.7. Western Blot Analysis

At the end of incubation, α-Syn expressed in the cells was analyzed by Western blot method. Cells were lysed in RIPA buffer containing PMSF, a protease inhibitor. Samples were sonicated and centrifuged, and pellets were discarded. The samples containing 25 mg of protein were loaded on SDS-PAGE and transferred to polyvinylidene difluoride (PVDF) membrane (Millipore, MA, USA). The PVDF membrane was then blocked in TBST (10 mM Tris-HCl, pH 7.4, 150 mM NaCl, 0.1% Tween-20, 5% skim milk) overnight. Blots were subsequently incubated with the rabbit anti-human α-Syn primary antibody (1:1000, CST, Boston, MA, USA, cat. no. 2628, Ct epitope) at 4 °C overnight. Peroxidase-conjugated secondary anti-rabbit antibody (1:2000, CST, USA, cat. no. 7074S) was used, and the bands were detected using the enhanced chemiluminescence reagents (ECL, Pierce, IL). β-actin (1:1000, CST, USA, cat. no. 4970T, Nt epitope) bands were used as a quantitative reference of other proteins. 

### 4.8. Immunofluorescence Microscopy

The cells were harvested from flasks and seeded on 0.01% poly-l-lysine coated glass slides in 12-well polystyrene plates (Corning Inc., NY) with 5 × 10^4^/mL cells in 1 mL medium per well. After incubation, the coverslips were fixed with 4% paraformaldehyde for 20 min at room temperature. Upon being washed with PBS, the permeabilization was done with 0.1% Triton X-100 in PBS for 5 min. Subsequently, 3% goat serum in PBS was applied to saturate unspecific epitopes for 30 min. The cells were incubated with a rabbit anti-human α-Syn primary antibody (1:250, CST, USA) for 1 h at room temperature. A secondary antibody (goat anti-rabbit IgG conjugated to Alexa Fluor 647, 1:250, CST, USA) was applied for 1 h. To stain nuclei and amyloid fibrils, DAPI and ThT were used for 15 min. After finally washed with PBS, coverslips were mounted in antifade medium and observed by a laser scanning confocal microscope (LSCM, C2+, Nikon, Japan).

### 4.9. Apoptosis of Transduced PC12 Cells Analyzed by FITC Conjugated Annexin V and PI 

After 24 h incubation, the cells were washed with PBS, resuspended in the blending buffer (an apoptosis detection kit containing annexin V-FITC and PI dyes, Dojindo Laboratorise, JP), and stained. Then, cells were incubated for another 15 min in the dark at 4 °C, and then analyzed by flow cytometry equipped with 488 nm argon laser light source, 515 nm bandpass filter for FITC-fluorescence, and 623 nm bandpass filter for PI-fluorescence (FCM, Beckman Coulter, Atlanta, GA, USA). A total of 10,000 events were acquired, and the cells were appropriately gated for analysis. Data were collected from three independent experiments. 

### 4.10. Measurement of Intracellular ROS 

ROS was measured using the oxidation-sensitive fluorescence probe, DCFH-DA (Sigma and Aldrich, USA, cat. no. D6883). At the end of incubation, 10 μM DCFH-DA was added to the cell and incubated for 30 min at 37 °C. DCFH-DA was deacetylated by intracellular esterase, and then further oxidized by ROS to form a fluorescent compound 2,7-dichlorofluorescein (DCF). Afterward, the cells were collected, and the DCF fluorescence was detected by FCM (excitation at 488 nm and emission at 525 nm). 10,000 events were acquired for each sample. Data were collected from three independent experiments.

### 4.11. Statistical Analysis

All data are presented as mean values ± SEM. SPSS 19.0 (SPSS, Inc., Chicago, IL, USA) was used for statistical analysis, and one-way ANOVA test was performed to analyze the statistical significance between two groups. A difference is considered to be statistically significant when the *p* value < 0.05.

## 5. Conclusions

In this paper, we demonstrated that EGCG can efficiently inhibit the formation of amyloid fibrils induced by Cu(II). EGCG coordinates with Cu(II) to form the Cu(II)/EGCG complexes, which functions better than EGCG alone. In addition, we demonstrated that EGCG can restrain Cu(II)-induced ROS, α-Syn overexpression and aggregation in PC12 cells, thus reducing Cu(II)-induced α-Syn toxicity. Overall, our studies prove that Cu(II)/EGCG complex would be a potential agent for the treatment of PD that would be even more efficient than EGCG alone.

## Abbreviations

CD	circular dichroism
DAPI	4’,6-diamidino-2-phenylindole
FACS	Fluorescence activated Cell Sorting
^1^H-NMR	^1^H nuclear magnetic resonance
LSCM	laser scanning confocal microscope
PBS	phosphate buffer saline
PD	Parkinson’s disease
ROS	reactive oxygen species
RIPA	Radio Immunoprecipitation Assay
SDS-PAGE	Sodium dodecyl sulfate - polyacrylamide gel electrophoresis
TEM	transmission electron microscopy
ThT	thioflavin T
